# Unraveling Melasma: From Epidermal Pigmentation to Microenvironmental Dysregulation

**DOI:** 10.3390/biology14101402

**Published:** 2025-10-13

**Authors:** Fang Miao, Jing Wan, Youwen Zhou, Ying Shi

**Affiliations:** 1 Department of Dermatology, Renmin Hospital of Wuhan University, Wuhan 430060, China; fangmiao@whu.edu.cn (F.M.); wan0311@whu.edu.cn (J.W.); 2 Department of Dermatology and Skin Science, University of British Columbia, Vancouver, BC V5Z 4E8, Canada; youwen.zhou@ubc.ca

**Keywords:** melasma, hyperpigmentation, epidermal–dermal crosstalk, microenvironment, immune dysregulation, photoaging, pathogenesis

## Abstract

**Simple Summary:**

Melasma is a chronic hyperpigmentation disorder that affects approximately 1% of the global population. It not only changes the appearance of the skin but also has a profound impact on the mental health of patients. In recent years, the focus of research has shifted from purely focusing on melanocytes to the complex interactions between the epidermis, dermis, blood vessels and the immune system. This article reviews key mechanisms from melanocyte activation and barrier dysfunction to extracellular matrix remodeling, angiogenesis and immune disorders. We also examined the roles of genetic susceptibility and the skin microenvironment in disease prolongation. This review integrates recent research results to build a unified pathogenesis framework and explore potential treatment strategies for multiple pathways.

**Abstract:**

Melasma is a chronic, acquired hyperpigmentation disease that occurs on light-exposed skin, especially in women of childbearing age. This common dyschromic disorder significantly impairs quality of life, yet treatments are unsatisfactory due to an incomplete understanding of its etiology. Its pathogenesis is multifactorial: ultraviolet (UV) radiation exposure, sex hormone fluctuations, and familial genetics are known triggers. Meanwhile, the persistence of focal hyperpigmentation suggests additional mechanisms beyond enhanced melanocyte activity. Emerging evidence highlights that melasma skin exhibits features of chronic photoaging: solar elastosis, basement membrane (BM) disruption and increased vascularity can be seen in the skin lesions. Senescent dermal fibroblasts under UV stress secrete melanogenic cytokines (e.g., SCF, HGF) that further stimulate melanocytes. In addition, melasma lesions harbor subclinical inflammation: infiltrates of CD4^+^ T cells, macrophages, and mast cells are visible, accompanied by elevated IL-17 and COX-2, implying an immune-driven component sustains pigment production. Collectively, these observations suggest that melasma behaves as a chronic inflammatory disorder of the skin microenvironment, rather than an isolated pigmentary defect. Concurrently, epidermal alterations such as barrier dysfunction and abnormal melanosome transport exacerbate melanin retention. In this review, by integrating these emerging insights into a unified pathogenic framework, we recognize melasma as a disorder of epidermal–dermal crosstalk and immune modulation, offering novel therapeutic perspectives for this recalcitrant condition.

## 1. Introduction

Melasma is a common acquired pigmentation disease, which is manifested by irregular pigmented patches on the skin (especially the face) in sun-exposed areas [1,2]. This chronic pigmentation disorder affects women particularly significantly, especially women of childbearing age and people of color with Fitzpatrick skin classification III–IV [1]. It is not only a cosmetic problem, but also closely related to diseases such as rosacea, atopic dermatitis, cancer, and nuclear cataracts [3,4], which seriously affect the quality of life of patients [5,6,7]. Although the incidence of melasma is high and brings a huge psychological burden to patients, current treatment strategies for melasma are often ineffective, and there are problems that pigmentation has not completely subsided and recurs frequently [8,9]. This therapeutic resistance underscores a fundamental gap in our understanding of its complex etiology and underlying pathogenic mechanisms. For decades, melasma was primarily viewed as excessive production and deposition of melanin in the epidermis caused solely by the hyperfunction of melanocytes [10]. However, the latest research evidence has piled up, breaking this perusal and revealing a network of pathogenic factors that is far more complex than melanocyte overactivity.

The pathogenesis of melasma is now widely believed to be caused by multiple factors, involving a complex interaction of genetic predisposition, hormonal fluctuations, and environmental triggers, most notably ultraviolet (UV) radiation exposure [11,12,13], as well as recently considered visible light exposure [14], heat exposure [15], alcohol intake [16], biorhythmic disorders [17], and exposure to hyperpigmenting drugs [18]. Although these factors have been recognized as the initiators or contributors to melasma, the persistent and often relapsing nature of local pigmentation suggests that deeper mechanisms continue to promote pigment production and retention. The latest research shifts the focus from simple epidermal pigment defects to a broader perspective, viewing melasma as a disorder of the entire skin microenvironment. This cognitive shift is supported by strong histological and molecular evidence, with studies showing that not only have significant structural and functional changes occurred in the epidermal layer, but also profound changes have occurred in the dermal layer and the epidermal–dermal junction [10,19,20].

The key characteristics of melasma-affected skin are the same as those of chronically photoaged skin. They all exhibit solar elastosis, a hallmark of dermal photodamage, accompanied by disruption of the basement membrane zone, increased vascularity, and an elevated number of mast cells in the dermis [12,21,22,23,24]. These dermal changes are by no means accidental but are increasingly recognized as critical contributors to the initiation and perpetuation of melanogenesis. For instance, senescent dermal fibroblasts, often a consequence of chronic UV exposure, have been shown to secrete an array of melanogenic factors, such as Stem Cell Factor (SCF) and Hepatocyte Growth Factor (HGF), which in turn strongly stimulate melanocytes to accelerate the production of melanin [12,25]. This highlights a crucial epidermal–dermal crosstalk that drives the hyperpigmentary phenotype.

Furthermore, growing evidence points towards a significant immune-driven component in melasma. Lesional skin frequently exhibits signs of subclinical inflammation, characterized by infiltrates of various immune cells, including CD4^+^ T cells, macrophages, and mast cells, accompanied by elevated levels of pro-inflammatory mediators such as Interleukin-17 (IL-17) and Cyclooxygenase-2 (COX-2). This suggests that melasma is not simply a pigmentary disorder but rather a chronic inflammatory state of the skin microenvironment, where immune cells and their secreted factors actively contribute to sustaining pigment production. At the same time, epidermal alterations, such as impaired epidermal barrier function and abnormal melanosomes transport, further exacerbate melanin retention within the skin [26,27]. These diverse elements—epidermal changes, dermal remodeling, and immune dysregulation—are not independent, but are intertwined through signaling pathways such as Wnt/β-catenin and PI3K/Akt and oxidative stress pathways, and are controlled by hormone fluctuations and environmental factors, jointly interpret the complex pathogenesis of melasma [5,25,28,29].

In this review, we conducted a literature search on PubMed, Web of Science Core Collection, and Google Scholar, using “melasma” as the topic term. Our objective was to integrate these emerging insights and build a comprehensive and unified framework for the pathogenesis of melasma. By moving beyond the traditional focus on melanocytes and embracing a holistic view that encompasses epidermal–dermal crosstalk, immune regulation, and broader microenvironment disorders, we seek to illuminate novel therapeutic perspectives for this challenging and stubborn disease. A deeper understanding of these multifaceted interactions is paramount for developing more effective and targeted treatment strategies that address the root causes of melasma, rather than just palliative pigmentation.

## 2. The Epidermal Landscape of Melasma: Beyond Melanocyte Hyperfunction

The predominant clinical manifestation of melasma is the presence of hyperpigmented macules and patches, which are fundamentally a result of increased melanin deposition in the skin. Historically, the primary focus of melasma research centered on the epidermal layer, specifically the melanocytes responsible for producing pigment. While enhanced melanocyte activity is still the core of melasma pathogenesis, recent investigations reveal that the epidermal landscape in melasma is far more complex than a simple increase in melanin production. It involves intricate cellular interactions, signaling pathways, and even alterations in the skin barrier function that collectively contribute to the characteristic hyperpigmentation.

### 2.1. Melanocyte Activity and Melanin Production

The most significant phenotypic feature of melasma is a surge in melanin content, which is clearly visible in both the epidermal and dermal layers of affected skin [5,21]. This heightened melanin content is a direct consequence of dysregulated melanogenesis, the biochemical process of melanin synthesis within melanocytes. While it is clear that melanocytes in melasma lesions are hyperfunctional, even protrude into the dermal layer to form so-called “pendulous melanocytes” [30], whether there is an actual increase in the number of melanocytes in melasma-affected skin remains controversial [21]. Some studies have shown an increase in its density, while others report normal numbers but with increased activity [31]. This disagreement just reveals the complexity of cellular changes in this disorder. Regardless of their absolute numbers, these melanocytes exhibit an enhanced capacity for melanin synthesis and transfer, leading to the characteristic hyperpigmentation.

**Figure 1:**
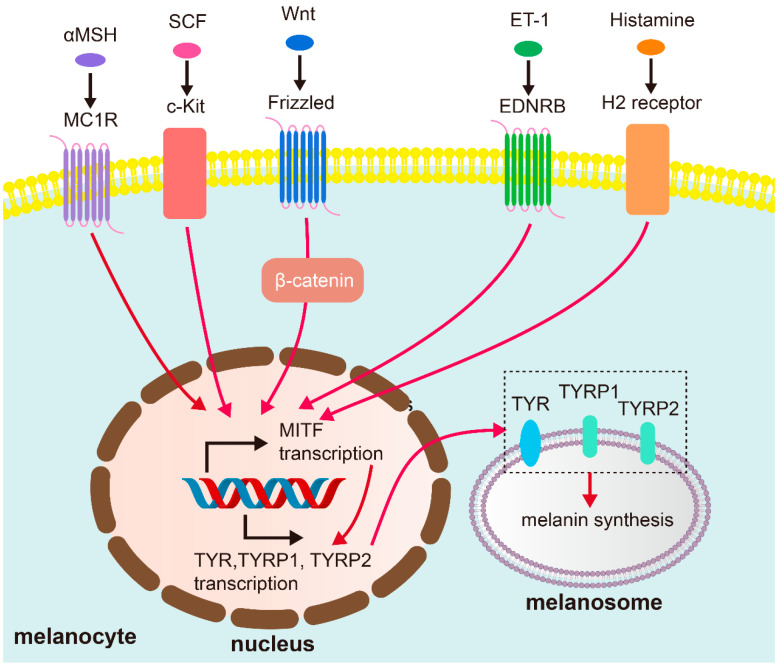
Representative pathways regulating melanogenesis in melasma. Mediate factors secreted by surrounding cells bind to receptors on the melanocyte membrane, activating MITF transcription, which in turn promotes the expression of TYR, TYRP1, and TYRP2, resulting in melanin synthesis.

The regulatory process of melanin production is complex and is dominated by numerous signaling networks that coordinate the activity of melanocytes and their interactions with surrounding cells. Factors such as α-melanocyte stimulating hormone (αMSH), SCF, KGF, and bFGF secreted by these cells bind to the receptor and eventually converge into the microphthalmia-associated transcription factor (MITF) (Figure 1), which transcribes the key enzymes that regulate melanin production–tyrosinase (TYR), tyrosinase-related protein 1 (TYRP1), and TYRP2 [32]. The core pathways regulating MITF include αMSH/melanocortin 1 receptor (MC1R), Wnt/β-catenin, PI3K/Akt, cAMP/PKA, and SCF/c-kit mediated signaling pathways [5]. Among them, when α-MSH binds to MC1R, it activates adenylate cyclase (AC), promotes cyclic adenosine phosphate (cAMP) production, and then activates protein kinase A (PKA). PKA phosphorylates the transcription factor CREB, thereby stimulating transcription of MITF [33]. The Wnt/β-catenin pathway, transduced by frizzled receptor, for instance, is crucial for melanocyte development and proliferation, and its dysregulation can lead to altered melanogenesis. Similarly, the PI3K/Akt pathway plays a significant role in cell survival, proliferation, and differentiation, including that of melanocytes, and its aberrant activation can contribute to hyperpigmentation. The cAMP/PKA pathway is a classic pathway that regulates melanin synthesis. Elevated cAMP levels usually enhance the activity of tyrosinase, the rate-limiting enzyme of melanin synthesis. Furthermore, the SCF/c-kit signaling pathway is particularly critical in the epidermal–dermal crosstalk. SCF, secreted by dermal fibroblasts and others, bind to c-kit receptors on the surface of melanocytes, stimulating their proliferation, migration, and melanogenic activity [5,25].

Another important paracrine mediator in melasma pathogenesis is endothelin-1 (ET-1), which is primarily secreted by keratinocytes after UV radiation. ET-1 expression is significantly elevated in melasma lesions compared to adjacent normal skin, with levels increased by approximately 32.8% [34]. ET-1 exerts its melanogenic effects by binding to endothelin receptor B (EDNRB) on melanocytes, activating downstream PKC, ERK1/2, and p38 MAPK signaling pathways, and ultimately upregulates MITF and melanin synthases [35]. The activation of these pathways is often triggered by external factors such as UV light, leading to excessive melanin production in melasma. Among them, ultraviolet B (UVB) radiation, as a powerful environmental inducement, not only upregulates the expression of multiple melanocyte-specific genes but also stimulates the release of key factors directly involved in melanin synthesis, thereby exacerbating the pigmentation phenotype [5]. The direct impact of UV light on melanocyte activity confirms the importance of sun protection in the treatment of melasma, emphasized by Morgado-Carrasco et al. [14].

### 2.2. Epidermal Barrier Dysfunction and Melanin Retention

In addition to the direct hyperactivity of melanocytes, there are other significant changes in the epidermal layer of melasma patients that lead to persistent and increasingly stubborn pigmentation. Recent research has shown that the skin barrier function in the melasma area is impaired and plays a pivotal role in its pathogenesis [27]. The skin barrier is mainly composed of the stratum corneum and its lipid matrix, which is crucial for maintaining skin moisture and resisting external attacks. Once this barrier is dysfunctional, it will intensify transdermal water loss, making the skin more susceptible to environmental stimuli, which may exacerbate the inflammatory process associated with melasma.

A refined lipidomic analysis of melasma lesions revealed significant abnormalities in the lipid composition of the skin surface [26,27]. Specifically, studies have shown that compared with normal skin, the content of total lipids, phosphatidic acid, phosphatidylserine and various ceramides (Cer) in the skin at the lesion site is significantly increased [27]. This increase in ceramides, particularly very long chain (VLC) (C20–C26) and ultra-long chain (ULC) (>C26) ceramide species, is hypothesized to be a compensatory mechanism aimed at maintaining the compromised skin barrier function [27]. However, the relationship between specific ceramide isoforms and melanocyte activation seems complex-for example, Cer[AH] was found to be negatively correlated with melanocyte activation in lesion areas, while certain lipids were underexpressed in dark lesions where melanocytes were highly activated [27]. These findings suggest that while the skin attempts to compensate for barrier dysfunction, the altered lipid profile itself might influence melanogenesis or melanosome transport.

Furthermore, the epidermal thickness of lesional skin in melasma has been observed to be higher compared to non-lesional skin [27]. This epidermal thickening and lipid abnormality together reveal that its pathological mechanism goes far beyond a simple disorder of pigment synthesis, but involves a broader imbalance of epithelial homeostasis. We specifically pointed out that “abnormal melanosome transport” is a key factor exacerbating the retention of melanin–melanosomes, which serve as melanin carriers, that need to be transported to the keratinocytes that make up the main body of the epidermis after being synthesized by melanocytes. Suppose this precise transport process is blocked, or the subsequent degradation of melanosomes in keratinocytes is abnormal. In that case, it leads to the accumulation of melanin in the epidermal layer, forming visible pigmentation. Increased melanocyte dendricity was observed in facial melasma biopsies, revealing the enhanced melanosome transport in this hyperpigmented disorder [36]. Recent review highlighted some specific proteins and signaling pathways, such as Rab27a, Myo5A, and melanophilin (Mlph) involved in regulating melanosome movement, and sustained the potential of targeting melanosome transport as an emerging strategy for pigmentation control [37]. Although the existing literature does not detail the specific mechanism of abnormal melanosomes transport in melasma, this finding once again confirms that epidermal dysfunction is the core link leading to the persistence of the disease. The interaction between lipid metabolism disorders, barrier dysfunction and melanosomes dynamics suggests that melasma treatment strategies should not only curb melanin synthesis, but also require a two-pronged approach to repair epidermal homeostasis and barrier function.

## 3. Dermal Remodeling and the Photoaging Connection

The traditional view of melasma as a purely epidermal pigmentary disorder has been significantly challenged by mounting evidence highlighting profound alterations within the dermal compartment. These dermal changes are not merely secondary effects but are increasingly recognized as pivotal contributors to the initiation, maintenance, and recurrence of melasma. What is intriguing is that many dermal changes are exactly the same as those of chronic photoaging skin, suggesting that melasma is inextricably linked to the broader photoaging process. This section delves into the key dermal modifications, including extracellular matrix abnormalities, basement membrane disruption, increased vascularity, and the role of senescent dermal fibroblasts. All of them highlight the core position of the dermal microenvironment in melasma pathogenesis.

### 3.1. Solar Elastosis and Extracellular Matrix (ECM) Abnormalities

One of the most common and significant pathological findings in the dermis of melasma-affected skin is the presence of solar elastosis [21,22,23]. Solar elastosis is a hallmark feature of chronic photodamage, which is manifested by the accumulation of a large number of distorted and thickened abnormal elastic fibers in the superficial layer of the dermis. This condition is a direct consequence of prolonged exposure to UV radiation (UVR), which induces the degradation of normal elastic fibers and collagen, followed by the aberrant synthesis of new, dysfunctional elastic material [28,38,39]. The presence of solar elastosis in melasma lesions undoubtedly closely links the disorder with photoaging, a process of premature skin aging induced by UVR [12,14,40].

The extracellular matrix (ECM) is a complex network of proteins and carbohydrates that not only supports tissue structure, but also plays an important role in cell adhesion, migration, proliferation and differentiation. The ECM in healthy skin is mainly composed of collagen (Types I, III, V) and elastin, which maintain the integrity and flexibility of tissue [28,41]. However, in photoaging skin and the ensuing melasma, this delicate balance is disrupted. UVR significantly affects the production and degradation of ECM components, resulting in a decrease in the total amount of collagen, but an increase in reactive oxygen species (ROS) and matrix metalloproteinases (MMPs) [28,40]. MMPs are a family of zinc-dependent endopeptidases capable of degrading various ECM proteins, including collagen, elastin, and proteoglycans [38,39]. During photoaging, UVR increases the level of MMPs such as collagenase (MMP-1, MMP-8, MMP-13) and gelatinase (MMP-2, MMP-9), thereby disintegrating collagen fibers and elastic fibers, which is the main culprit of skin wrinkles and loss of elasticity [38,39]. Additionally, the effects of ECM degradation by MMPs, ECM abnormalities, and solar elastin on the pathogenesis of melasma are complex. Damage to dermal ECM may alter the physical and biochemical signal exchange between dermal cells and melanocytes, thereby affecting melanogenesis. In addition, damaged ECM components can also trigger an inflammatory response, resulting in an overall imbalance of the skin microenvironment. The latest research has also revealed the key role of exosomes (tiny extracellular vesicles) in skin photoaging. Stem cell-derived exosomes have demonstrated the potential to repair skin physiological functions and revitalize damaged skin tissue by reducing MMP expression and promoting collagen and elastin production [42]. Given that melasma is closely related to photoaging, targeted regulation of ECM integrity and MMP activity may become a new treatment idea.

### 3.2. Basement Membrane (BM) Disruption

Another critical structural alteration in melasma is the disruption of the basement membrane (BM) [21,22,23]. The BM is a specialized ECM structure located at the epidermal–dermal junction, serving as a crucial interface that mediates communication and adhesion between keratinocytes of the epidermis and fibroblasts and other cells of the dermis. It can not only act as a selective barrier, regulating the passage of molecules and cells, but also provide structural support for the epidermis. While the incidence of BM disruption in melasma has been described as variable, its presence is consistently noted in histological examinations [22].

The integrity of the BM is critical to maintaining skin homeostasis. Disruption of the BM in melasma may have a profound impact on pigment regulation. A compromised BM may allow for the “dropping” of melanin from the epidermis into the dermis, where it is then phagocytosed by dermal macrophages, leading to the characteristic dermal component of melasma (melanophages) [5]. This phenomenon contributes to the persistent and often treatment-resistant nature of dermal melasma.

In addition, structural disorders of the BM can also alter paracrine signaling between melanocytes and dermal cells. BM contains a variety of growth factors and cytokines that affect the behavior of melanocytes, and their structural destruction may expose melanocytes to an abnormal dermal microenvironment, thereby activating their function or affecting melanosomes transport. The exact mechanism of BM destruction in melasma is likely related to chronic UV exposure and related ECM degradation processes, including the activity of MMPs that degrade BM components.

If the specific factors leading to BM destruction can be clarified and strategies to repair its integrity developed, it may open up new avenues for treating melasma.

### 3.3. Increased Vascularity (Angiogenesis)

The dermis of melasma-affected skin consistently exhibits an increase in vascularity, characterized by an elevated number and size of blood vessels [23,43]. This increased vascularity, or angiogenesis, is now recognized as a pivotal dermal factor contributing to melasma pathogenesis, although the disorder is mainly manifested by epidermal hyperpigmentation [22]. Studies have shown that the existence of abnormal vascular endothelial cells is closely related to the pathogenesis of the disease [25].

Newly formed or dilated blood vessels in melasma lesions do not stand by; they can fuel and intensify the pigmentation process. Endothelial cells on the inner wall of blood vessels can secrete a variety of melanogenic factors and inflammatory mediators, directly or indirectly stimulating melanocytes. Particularly critical are SCF and Vascular Endothelial Growth Factor (VEGF) [25]. As mentioned earlier, SCF stimulates melanocyte proliferation and melanin synthesis through c-kit receptors, and is actually a key driver of melanin production [5,25]. VEGF is a potent pro-angiogenic factor that promotes the formation of new blood vessels. Its elevated presence in melasma lesions suggests an active angiogenic process. On vascular endothelial cells, VEGF receptors are present, facilitating melanogenesis by inducing the production of ET-1, which subsequently activates MITF phosphorylation and increases tyrosinase levels [44]. The expanded vascular network is like a hub extending in all directions, continuously transporting these melanogenic factors and inflammatory mediators to the epidermis-dermis junction, making melanocytes continue to be excited, and ultimately causing overall pigmentation.

For example, studies have found that 590 nm LED irradiation can significantly inhibit the migration of human microvascular endothelial cells (HMEC-1), lumen formation (a marker of angiogenesis), and the expression of VEGF and SCF [25]. The mechanism is that 590 nm LEDs play a role by blocking phosphorylation of the AKT/PI3K/mTOR signaling pathway-this pathway is important in cell growth, proliferation and angiogenesis [25,28,45]. This inhibitory effect was partially reversed when the pathway agonist insulin-like growth factor 1 (IGF-1) was used, further verifying the mechanism [25]. Clinical pilot studies have shown that amber LED phototherapy can significantly improve facial erythema (redness due to increased vascularity) and pigmentation in melasma patients [25]. Tranexamic acid improves melasma by down-regulating ET-1 expression in dermal microvascular endothelial cells, confirming the therapeutic value of this pathway [46]. These findings reveal the therapeutic potential of targeting vascular components, suggesting that inhibiting angiogenesis and endothelial cell melanotropin secretion can be a two-pronged approach while alleviating erythema and stain problems.

### 3.4. Senescent Dermal Fibroblasts

Dermal fibroblasts, as the main cells of the dermal layer, play an important role in maintaining skin structure and function by synthesizing ECM components [47]. However, under conditions of chronic stress, particularly UVR, these cells fall into a state of senescence, an irreversible cell cycle arrest accompanied by a unique secretory phenotype known as the senescence-associated secretory phenotype (SASP) [12,48,49,50]. Senescent dermal fibroblasts are increasingly recognized as significant contributors to the pathogenesis of melasma.

In melasma lesions, senescent dermal fibroblasts, often induced by chronic UV exposure, secrete a variety of melanogenic factors that directly stimulate melanocytes [12]. Prominent among these factors are SCF, Keratinocyte Growth Factor (KGF) and Hepatocyte Growth Factor (HGF) [12,51]. As mentioned earlier, SCF is not only a powerful melanocyte activator, but also leads to the recruitment and proliferation of c-KIT^+^ mast cells [52]. KGF induces an increase in the transfer and uptake of melanosomes from melanocytes to keratinocytes through phagocytosis. HGF also promotes melanocyte proliferation and melanin synthesis. In addition, VEGF secreted by these cells can directly stimulate neovascularization, and MMPs degrade the collagen matrix and disrupt the BM [52]. The continuous secretion of these factors by senescent fibroblasts creates a persistent pro-melanogenic microenvironment within the dermis, contributing to the recalcitrant nature of melasma. Furthermore, the gene expression profile of senescent fibroblasts in melasma lesions comprised pro-inflammatory, pro-melanogenic, and tissue repair deficit-related factors, which can induce damage to the upper dermis and support the focal pigmentary phenotype in melasma [53]. This reveals a key mechanism of epidermal–dermal interaction: dysfunctional dermal cells actively drive epidermal hyperpigmentation [52].

On the other hand, the connection between fibroblast senescence and photoaging is well-established. Photoaging, induced by UVR, leads to direct damage to DNA, exacerbates oxidative stress, and activates cell signaling pathways that lead to skin integrity destruction and cellular senescence [28,40]. Senescent cells accumulate in photoaged skin, triggering chronic low-grade inflammation known as “inflammaging” [50]. This chronic inflammation, driven by senescent cells, may further deteriorate the pro-melanogenic environment. Studies on UVB-induced photoaging models have shown that UV exposure leads to characteristics of photoaging in human dermal fibroblasts, including changes in m6A RNA modification and the regulation of microRNAs like miR-100-3p, which can affect fibroblast senescence [48]. Furthermore, UVA-induced photoaging in fibroblasts involves mitochondrial oxidative stress and the activation of the PI3K/AKT/mTOR signaling pathway, which can be attenuated by compounds like metformin [45]. Resveratrol, another natural compound, has been shown to protect against UVA-induced photoaging in human skin fibroblasts by activating autophagy and regulating the AMPK pathway, reducing ROS production, and inhibiting apoptosis [54]. These findings reveal the complex molecular mechanisms behind fibroblast senescence during photoaging and its direct relevance to melasma. The persistence of senescent fibroblasts in the dermal microenvironment, continuously secreting melanogenic factors, provides a compelling explanation for the chronic and relapsing nature of melasma, suggesting that targeting fibroblast senescence could be a promising therapeutic strategy [52].

### 3.5. Sebaceous Glands

Although the face suffers from sun exposure all year round, melasma occurs in specific areas where sebaceous glands (SGs) are rich, such as the cheekbones, forehead, and upper lip [55]. SGs are epithelial appendages typically associated with hair follicles, which constitute the niche for melanocyte stem cell residence [56]. Previous studies have shown that active factors released by SZ95 sebaceous cells can stimulate the proliferation of human epidermal melanocytes and promote the formation of dendrites [57]. Its ability to significantly maintain epidermal melanocyte activity was further revealed through the 3D-SeboSkin model (skin explants co-cultured with SZ95 sebocytes) [58]. Sebaceous cells irradiated by UVA not only produce and upregulate the factors α-MSH, EDN1, SCF, and b-FGF, but also promote melanogenesis and activate dermal inflammation and aging [59]. What is particularly intriguing is that melanocytes are also hidden in human sebaceous glands [60]. These findings conclusively suggest that sebaceous glands are directly or indirectly involved in the pathogenesis of melasma.

## 4. Immune Dysregulation and Chronic Inflammation

In addition to structural and cellular changes in the epidermis and dermis, increasing evidence suggests that immune disorders and chronic subclinical inflammation play a pivotal role in the pathogenesis of melasma. This paradigm shift in cognition has made us realize that melasma is not merely an isolated pigmentary defect, but a chronic inflammatory disorder of the skin microenvironment, where immune cells and their mediators actively contribute to sustaining pigment production. The presence of specific immune cell infiltrates and elevated inflammatory markers in melasma lesions strongly supports this view.

### 4.1. Mast Cell Involvement

Mast cells are crucial components of the immune system, strategically localized in the perivascular areas of the dermis, making them highly responsive to changes in the microenvironment [61]. In melasma, an increased number of mast cells in the dermis is a consistent histological finding, indicating their important role in the disease’s pathogenesis [12,21,22,23]. Mast cells are versatile immune cells capable of performing both pro-inflammatory and anti-inflammatory roles, depending on the context and the specific stimuli they encounter [61]. Their ability to release a wide variety of preformed and newly synthesized effector molecules upon activation makes them key players in immune regulation and tissue responses.

Upon degranulation, mast cells release a series of powerful “cocktails” of mediators, including histamine, tryptase, cytokines, and growth factors [61,62]. These mediators can not only directly stimulate melanocytes but also influence the surrounding dermal microenvironment, thereby contributing to the promotion of melanogenesis. For instance, mast cell-derived mediators can induce endothelial inflammatory responses, which are synergistically amplified by bacterial toxins, potentially contributing to the increased vascularity observed in melasma [61]. Furthermore, mast cells exhibit intimate interactions with sensory nerves, contributing to neurogenic inflammation in the skin [62,63].

### 4.2. T Cells and Macrophages

Melasma lesions are characterized by infiltrates of various immune cells, including CD4^+^ T cells and macrophages, alongside mast cells. These cellular infiltrates signify a subclinical inflammatory process that actively contributes to the sustained pigment production. T cells are central orchestrators of immune responses in the skin, and their specific subpopulations dictate the nature of inflammation [64]. In the context of skin inflammation, T cell-mediated diseases can be classified based on the dominant T cell subset involved, such as T1, T2, T17/T22, or Treg cell-dominated responses [64]. While specifically mentioning CD4^+^ T cells, the elevated IL-17 levels observed in melasma lesions strongly suggest the involvement of T helper 17 (Th17) cells, which are known producers of IL-17. Th17/T22 cell-dominated responses are characteristic of inflammatory skin diseases like psoriasis [64]. IL-17 is a potent pro-inflammatory cytokine that can influence keratinocyte behavior and contribute to the inflammatory milieu. For instance, IL-17D has been shown to induce inhibition of the RNA helicase DDX5 in keratinocytes, which in turn amplifies IL-36R-mediated skin inflammation by favoring the production of membrane-bound, intact IL-36 receptor at the expense of soluble IL-36R [65]. This mechanism, described in atopic dermatitis and psoriasis, highlights how specific cytokines can modulate keratinocyte responses to perpetuate inflammation, potentially contributing to the inflammatory component of melasma.

Macrophages are another critical immune cell type found in melasma lesions. These versatile phagocytic cells play pivotal roles in orchestrating inflammation, fibrosis, and wound repair [66]. Macrophages exhibit plasticity, transitioning between pro-inflammatory (M1-like) phenotypes, which are prevalent early post-injury, and anti-inflammatory (M2-like) phenotypes, which appear later to modulate skin repair and wound closure [66]. In the context of melasma, the presence of macrophages, particularly those with pro-inflammatory characteristics, could contribute to the sustained inflammatory environment. Macrophages secrete a wide array of cytokines, chemokines, and growth factors that can directly or indirectly influence melanocyte activity and the overall dermal microenvironment. For example, the elevated COX-2 levels observed in melasma lesions suggest increased prostaglandin synthesis, which can be mediated by macrophages and other inflammatory cells, and which are known to stimulate melanogenesis.

Furthermore, the interplay between immune cells and other skin components is crucial. For instance, Gasdermin E (GSDME)-mediated pyroptosis, a form of programmed cell death, is induced in keratinocytes of UVB-challenged skin. GSDME deficiency has been shown to aggravate UVB-induced skin inflammation by enhancing the recruitment and activation of neutrophils, suggesting a restrictive role for GSDME in controlling inflammation [49]. While the direct link to melasma is not explicitly stated in this context, it illustrates how keratinocyte responses to UV can influence immune cell infiltration and the inflammatory cascade, which is highly relevant to melasma’s pathogenesis. The concept of mechanotransduction, where cells perceive and interpret mechanical stimuli, also plays a role in skin inflammation. Dysregulated mechanotransduction can provoke inflammatory processes, as seen in the Koebner phenomenon, and involves interactions between various cells, including keratinocytes, Langerhans cells, endothelial cells, and mast cells [67]. While more studied in psoriasis, this mechanism could also contribute to the inflammatory component of melasma, especially given the structural changes in the ECM.

### 4.3. Neurogenic Inflammation and the Skin Microenvironment

While less extensively studied in melasma compared to other inflammatory skin conditions, the concept of neurogenic inflammation offers another layer of complexity to the disease’s pathogenesis. The skin is richly innervated, and nerve endings can release neuropeptides (e.g., substance P, calcitonin gene-related peptide (CGRP)) that act as pro-inflammatory mediators [63]. These neuropeptides can interact with various skin cells, including keratinocytes, endothelial cells, and immune cells like mast cells, to propagate inflammatory responses [62,63]. A previous study found that fibroblasts from the melasma lesion and perilesional skin secreted more nerve growth factor (NGF)-β than those in normal skin, followed by increased melanogenesis [68]. Also, direct evidence shows that melasma lesions markedly express nerve growth factor receptor (NGFR) and neural endopeptidase (NEP) than nonlesional skin, suggesting that neuroactive molecules, including NGF, may directly affect the microenvironment around melanocytes through a NGFR immunoreactive nerve fiber pathway, and higher levels of NEP have an essential role in the regulation of melanogenesis [69].

The activation of transient receptor potential vanilloid (TRPV) ion channels, for instance, can lead to the release of CGRP and substance P, contributing to cutaneous neurogenic inflammation [63]. Immune cells in the skin also express TRPV1, and their activation directly affects their function, mediating communication between sensory nerve endings and skin immune cells, thereby increasing the release of inflammatory mediators [63].

House dust mites, for example, have been shown to activate nociceptors (neuropeptide-producing sensory neurons), which then induce mast cell degranulation via substance P and activation of the MRGPRB2 receptor on mast cells, driving type 2 skin inflammation [70]. While this specific mechanism is described in allergic skin diseases, it illustrates the principle of neuro-immune interaction involving mast cells that could be relevant in melasma’s inflammatory component. The increased morphological contacts between mast cells and sensory nerves in chronic inflammatory skin diseases like psoriasis and atopic dermatitis further support the essential role of this communication in driving inflammation and pruritus [62]. In the context of melasma, this neuro-immune axis could contribute to the sustained inflammatory environment that promotes melanocyte activity.

Understanding these molecular mechanisms could pave the way for novel treatments targeting the neuro-inflammatory axis in melasma. A recent split-face, randomized control study showed that botulinum toxin A helps to treat and prevent recurrence of melasma, with the possible mechanism of inhibiting the release of vasoactive substances, such as substance P and CGRP, from sensory nerves [71]. This finding further corroborates the feasibility of this therapeutic approach.

In essence, melasma is increasingly viewed as a chronic inflammatory disorder of the skin microenvironment, where subclinical inflammation, driven by immune cell infiltrates and potentially neurogenic mechanisms, creates a persistent pro-melanogenic milieu. This inflammatory cascade, often initiated and exacerbated by UV radiation, contributes significantly to the recalcitrant nature of the hyperpigmentation, highlighting the need for therapeutic strategies that address not only melanin production but also the underlying inflammatory processes.

## 5. Key Modulating Factors in Melasma Pathogenesis

The pathogenesis of melasma is not merely a sum of isolated epidermal, dermal, and immune alterations; instead, these diverse elements converge through intricate and interconnected signaling networks. These pathways are modulated by a variety of internal and external factors, including UVR, hormonal fluctuations, genetic predisposition, and recently recognized visible light (VL), exposure to medications, ultimately orchestrating the complex hyperpigmentary phenotype. Understanding these complex crosstalks is paramount for developing comprehensive and effective therapeutic strategies.

### 5.1. Ultraviolet (UV) Radiation: The Primary Environmental Trigger

Ultraviolet (UV) radiation, particularly from sun exposure, is unequivocally recognized as the most significant environmental trigger and aggravating factor for melasma [5,11,12,14]. Its profound impact on skin cells initiates a cascade of events that directly and indirectly contribute to hyperpigmentation. UV radiation, especially UVB, directly upregulates the expression of several melanocyte-specific genes and stimulates the release of key factors that participate in melanin synthesis, thereby enhancing melanocyte activity and melanin production [5]. This direct melanogenic effect is a primary reason why photoprotection is a cornerstone of melasma management [14].

Beyond its direct effect on melanocytes, UV radiation induces significant damage to skin cells through various mechanisms. It causes direct damage to DNA, generates reactive oxygen species (ROS) leading to oxidative stress, and activates numerous cell signaling pathways that contribute to the loss of skin integrity and cellular dysfunction, characteristic of photoaging [28,40]. Oxidative stress, an imbalance between ROS production and antioxidant defenses, is a critical component of UV-induced skin damage and is strongly implicated in melasma pathogenesis [5,28,72]. ROS can directly degrade essential biological macromolecules like proteins, DNA, and lipids, impairing cell metabolism and survival. Furthermore, ROS mediates the activation of crucial signaling pathways, including MAPK, JAK/STAT, PI3K/AKT/mTOR, NF-κB, Nrf2, and SIRT1/FOXO, which in turn affect cytokine release and enzyme expression, perpetuating inflammation and cellular senescence [28].

The role of UV in inducing photoaging is well-documented. Resveratrol, a natural polyphenol, has been extensively studied for its photoprotective effects against UVB-induced photoaging. Its mechanism of action involves reducing the expression of matrix metalloproteinases (MMPs), which degrade collagen, and suppressing inflammatory factors. Resveratrol achieves this by inhibiting ROS-mediated MAPK and COX-2 signaling pathways, balancing oxidative stress through the Nrf2 signaling pathway, and inducing antiapoptotic effects by inhibiting caspase activation. It also exerts antioxidant and antiapoptotic effects by targeting VEGF-B [73]. Similarly, resveratrol has been shown to activate autophagy and protect against UVA-induced photoaging in human skin fibroblasts and mouse models by regulating the AMPK pathway, reducing ROS production, and inhibiting apoptosis [54]. These findings underscore the profound impact of UV on skin cellular processes and the potential of antioxidants and pathway modulators to counteract these effects.

Further insights into UV-induced photoaging reveal the involvement of specific molecular players. For instance, METTL14, an m6A RNA methyltransferase, affects UVB-induced human dermal fibroblast photoaging via miR-100-3p biogenesis in an m6A-dependent manner, highlighting the role of epigenetic modifications in photoaging [48]. Another study identified that 5′-tiRNA-His-GTG expression levels are significantly elevated in photoaging cell models and mouse skin, and its overexpression induces cellular senescence by targeting nuclear pore proteins 98, which further activates the JNK signaling pathway [74]. These findings demonstrate the intricate molecular responses of skin cells to UV exposure, which contribute to the chronic changes observed in melasma.

### 5.2. Hormonal Fluctuations: Endogenous Modulators

Melasma, also referred to as the “mask of pregnancy” or “chloasma,” commonly affects women during pregnancy, suggesting a possible link between sex hormones and its development. Although hormonal fluctuations—especially those associated with pregnancy, oral contraceptive use, and hormone replacement therapy—are recognized as triggers or exacerbating factors for melasma, emerging evidence suggests a more complex interaction [5,11,12].

An in vitro study indicates that estrogen alone does not directly induce hyperpigmentation but acts synergistically with UVB radiation [75]. Clinical, estrogen associated with UVR can recapitulate the specific melanosome distribution observed in caucasoid melasma [76]. Moreover, estrogen may contribute to sustained pigmentation by increasing vascularization and stimulating the secretion of endothelin-1 (ET-1), which can further promote melanogenesis. Unlike in females, testosterone possibly plays a role in the development of melasma in males [77]. A literature review also highlights that the relationship between hormonal changes and melasma remains unclear in both women and men [78].

Recently, a case–control study on the United States population shows that exposure to exogenous hormones is a significant risk factor across all races, highest in Whites [18]. All synthetic progestins have been associated with an increased risk of melasma, with fourth-generation progestins posing the highest risk and third-generation the lowest [79]. A population-based cohort study also suggests that the risk of melasma is highest with combined oral contraceptives (COCs), which contain both estrogen and progestin, followed by progestin-only contraceptives (POCs). In contrast, hormonal intrauterine devices (hIUDs), which deliver hormones locally at lower systemic levels, are associated with the lowest risk [80]. Furthermore, melasma has been observed to improve spontaneously in patients who switched from combined oral contraceptives to levonorgestrel-releasing intrauterine devices, suggesting that systemic—rather than local—effects of estrogen may be responsible for melasma onset [81]. In addition, oxytocin (OXT) significantly increases normal human cultured melanocytes’ proliferation and migration and increases melanin production by upregulating melanogenesis-related MITF, tyrosinase, TYRP-1, and TYRP-2 expression [82].

Recent network pharmacology and molecular dynamics studies investigating potential melasma treatments have highlighted the involvement of estrogen receptors (ESR1 and ESR2) and the prolactin signaling pathway in melasma pathogenesis [29]. These studies suggest that compounds interacting with these receptors could regulate the disease. For example, specific Cassipourea metabolites have shown significant binding effects on ESR1 and ESR2, suggesting their potential as modulators in melasma treatment [29]. This indicates that hormonal influences extend beyond simply triggering melanocyte activity to involve complex signaling pathways that can be targeted therapeutically.

These findings support the hypothesis that, at least in women, sex hormones are indeed relevant to melasma risk, although the precise mechanisms and contributing factors continue to be explored.

### 5.3. Genetic Predisposition: The Underlying Susceptibility

Genetic susceptibility is another crucial factor contributing to the pathogenesis of melasma [5,12]. A significant proportion of melasma patients report a family history of the condition, suggesting a strong genetic component [13]. While melasma is not a monogenic disorder, specific gene polymorphisms have been associated with an increased risk (Summary in Table 1). For instance, a study on Javanese women found that the heterozygote Val92Met polymorphism of the Melanocortin-1 Receptor (MC1R) gene was significantly associated with the incidence of melasma [13]. MC1R plays a critical role in regulating skin pigmentation, primarily by switching melanin production from pheomelanin (red/yellow pigment) to eumelanin (brown/black pigment). Variants in MC1R can alter its function, influencing an individual’s skin phototype and susceptibility to UV-induced damage and pigmentary disorders. This finding, along with sun exposure and family history, is identified as a risk factor, underscoring the gene-environment interaction in melasma [13]. While the Arg163Gln genotype of MC1R was not found to be a risk factor in this specific population, the association with Val92Met highlights the importance of genetic background in predisposing individuals to melasma.

Restriction fragment length polymorphism polymerase chain reaction (RFLP PCR) assay investigated 45 female melasma patients in Egypt, and confirmed that Vitamin D Receptor Gene Polymorphism (TaqI) polymorphism, especially the presence of (t) allele and (tt) genotype, is associated with melasma [83]. A pilot study in African women with melasma indicated that the rs1042602 single-nucleotide polymorphisms (SNP) in the *TYR* gene showed a strong association with melasma, with the AA genotype conferring a markedly increased risk. The rs1129038 SNP in the *HERC2* gene and the rs1426654 SNP in the *SLC24A5* gene revealed significant genetic variations between groups in women of African descent [84]. Further research into other genetic variants and their interactions with environmental and hormonal factors is crucial for a comprehensive understanding of melasma susceptibility.

### 5.4. Recently Recognized Factors

Besides UV, visible light, especially high-energy visible light (HEVL), plays a key role in melasma pathophysiology, particularly in darker-skinned individuals through increasing ROS generation, MMPs expression, collagen degradation, and inducing indirect DNA damage [14]. Acknowledging the central role of visible light in melanogenesis, some dermatologists propose that changing the terminology from “sunscreen” to “light protection” [85,86].

Oral contraceptives and phototoxic drugs are common triggers of melasma; various other medications can also promote melanin synthesis, leading to the clinical manifestation of melasma [87]. As their metabolism generates ROS, some antibiotics such as metronidazole and clarithromycin are linked to melasma [87]. Oral retinoids such as isotretinoin [88] or alitretinoin [88] also aggravate melasma, with the underlying mechanisms of hormonal imbalances or directly enhancing TYR expression, respectively. Likewise, some drugs targeting melanogenesis-associated genes or signal factors may trigger melasma. In brief, exposure to hyperpigmenting drugs, including doxycycline, chloroquine, and cyclophosphamide, conferred a significant risk of melasma across all races, highest in Blacks and Hispanics [18].

A cross-sectional multicentric study in India suggests that the duration of cooking fire/occupational heat exposure may be linked to the severity of melasma [15]. A retrospective case–control study in China highlights the alcohol intake as a novel triggering factor of melasma [16]. Some studies reveal the correlation between biorhythmic disorders and the initiation and severity of melasma [17].

As discussed above, the complicated pathogenesis of melasma, triggered by external stressors like UV, VL, drugs, and internal factors like hormones and genetics, creates a complex and dynamic microenvironment in this disease. Importantly, these triggers are not isolated: UV radiation amplifies estrogen signaling, genetic predispositions modulate oxidative stress responses, and hormones influence angiogenesis and immune activity. These interconnected networks converge to sustain a pro-pigmentary microenvironment. By underscoring the multifactorial nature of the disease, we highlight the need for therapeutic approaches that target multiple pathways simultaneously to achieve sustained improvement. Recognizing melasma as a disorder of epidermal–dermal crosstalk and immune modulation, orchestrated by these complex networks, offers novel and promising therapeutic perspectives for this recalcitrant condition.

## 6. Therapeutic Implications and Future Directions

The evolving understanding of melasma’s pathogenesis, particularly the shift from a purely epidermal view to one encompassing dermal microenvironmental dysregulation and immune modulation, has significant implications for therapeutic strategies. Traditional treatments often focus on reducing melanin production or removing existing pigment, but their limited efficacy and high recurrence rates highlight the need for approaches that address the underlying multifactorial mechanisms [5,8].

### 6.1. Photoprotection as a Cornerstone

Regardless of the specific therapeutic approach, tailored photoprotection remains the cornerstone of melasma management [14]. Given that UV radiation is a primary trigger and exacerbating factor, comprehensive photoprotection, including broad-spectrum sunscreens and physical barriers, is essential for preventing recurrence and enhancing treatment outcomes. The understanding that visible light (VL) also influences melasma further emphasizes the need for advanced photoprotective strategies that block a wider spectrum of light [23,89].

### 6.2. Advanced Tyrosinase Inhibition Strategies

Tyrosinase (TYR) remains a primary target for depigmenting agents due to its critical role in melanin synthesis, catalyzing the rate-limiting steps in the pathway [90,91]. TYR inhibitors can be included in three groups: true inhibitors, specific TYR inactivators and alternative substrates. The compounds that can act as alternative substrates should not be used since TYR can act on them, generating o-quinones other than o-dopaquinone, diverting the melanin biosynthesis pathway [92]. While many existing agents directly inhibit tyrosinase activity, newer strategies are exploring more nuanced ways to disrupt its function or the melanosome environment.

**Figure 2:**
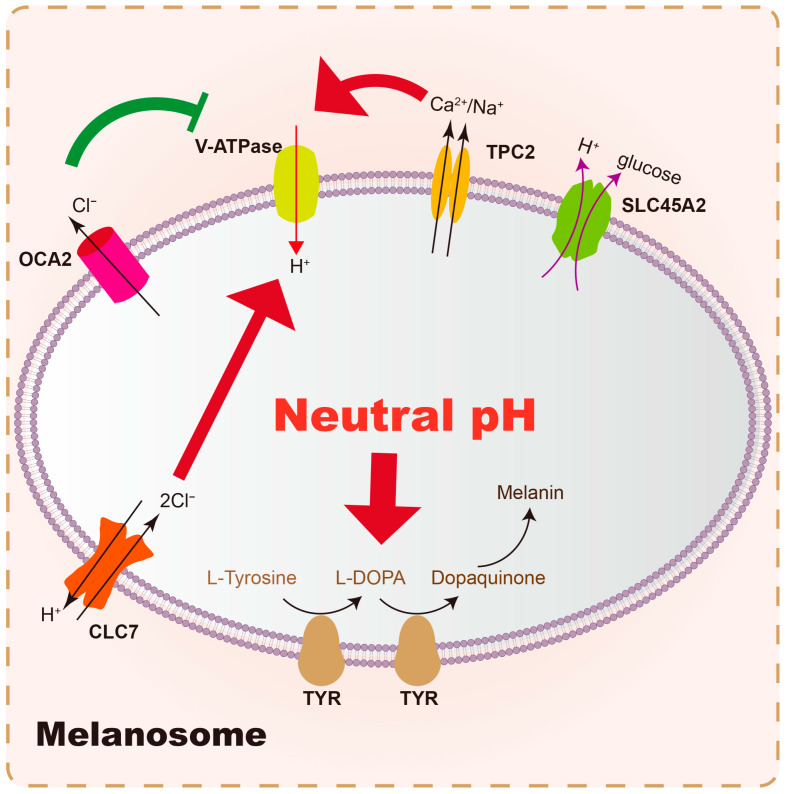
Main membrane proteins involved in intramelanosomal pH homeostasis. Vacuolar-type ATPase (V-ATPase) pumps H^+^ into melanosomes and serves as the primary driver of melanosome acidification. In contrast, the SLC45A2 protein elevates the internal pH of melanosomes by pumping H^+^ out of these organelles. Additionally, the OCA2 protein exports Cl^−^ from the melanosomal lumen, which reduces the driving potential of the V-ATPase, thereby leading to an increase in the intramelanosomal pH. On the other hand, TPC2 and CLC7 enhance the acidifying activity of V-ATPase by mediating the efflux of Ca^2+^/K^+^ and the influx of Cl^−^, respectively. Ultimately, tyrosinase exerts its optimal catalytic activity in the slightly neutral pH microenvironment maintained collectively by the aforementioned membrane proteins, facilitating the production of melanin.

Melanosomes are specialized organelles within melanocytes where melanin synthesis and storage occur. The internal environment of melanosomes, particularly their pH, is crucial for optimal tyrosinase activity. The near-neutral pH (~6.8) of the mature melanosome is thought to provide a favorable environment for TYR [93]. The dynamic maintenance of pH in melanosomes is believed to be controlled by several membrane proteins. Briefly, the central melanosomal anion channel mediated by OCA2 expression transports Cl^−^ into the cytoplasm to make the melanosome membrane voltage more negative, thereby decreasing V-ATPase-mediated H^+^ transport and luminal acidity, which enhances the activity tyrosinase and melanin production [94]; TPC2 mediated Ca^2+^/Na^+^ efflux, along with CLC7/OCA2 mediated H^+^ and Cl^−^ flux, regulates the activity of V-ATPase to maintain luminal ionic homeostasis and pH [95]. (Summary in Table 2). OCA2 and SLC45A2 are presumed to be positive pH regulators, while V-ATPase and TPC2 are considered to be negative pH regulators (Figure 2). Strategies aimed at melanosome acidification would likely involve skin whitening by inhibiting tyrosinase activity [96,97].

These strategies represent a significant advancement in tyrosinase inhibition, moving from direct enzyme binding to modulating the cellular and organellar environment critical for its function. Although their specific mechanisms remain to be fully elucidated, we emphasize their significance as promising emerging targets for future melasma therapies, providing a more precise and potentially more effective approach to regulating melanin synthesis.

### 6.3. Targeting Vascularity and Inflammation

The recognition of increased vascularity and subclinical inflammation as key drivers suggests that therapies targeting these aspects could be highly beneficial. Pulsed-dye laser (PDL), which targets vascular lesions, has shown promise, especially in patients with visible capillaries, when combined with other laser treatments [104]. Furthermore, the investigation into 590 nm LED irradiation, which inhibits angiogenesis and the secretion of pro-melanogenic factors by endothelial cells via the AKT/PI3K/mTOR pathway, represents a novel therapeutic option that directly addresses the vascular component of melasma [25]. This approach offers a comparative advantage over purely pigment-targeting therapies by addressing the root cause of sustained melanogenesis.

### 6.4. Modulating Oxidative Stress and Photoaging

Given the strong link between melasma and photoaging, strategies that mitigate oxidative stress and reverse photoaging-related damage are promising. Antioxidants, whether topical or systemic, can help neutralize ROS and protect skin cells [28,40]. Vitamin C, silymarin, and niacinamide have all demonstrated promising potential for reducing pigmentation in melasma. Vitamin C displayed significant effects in meta-analysis [105]. Compounds like resveratrol and luteolin, by modulating oxidative stress pathways and inhibiting inflammatory factors and MMPs, offer potential for both prevention and treatment of melasma by addressing the photoaging component [71,106]. As a hormone that regulates circadian rhythms, melatonin also possesses antioxidant properties. When used alone or in combination with other therapies, it can help improve the appearance of melasma [107]. Further research into the role of endogenous antioxidants like bilirubin in melasma might also reveal novel therapeutic targets [72]. The use of metformin to suppress the PI3K/AKT/mTOR pathway and inhibit mitophagy in photoaging also presents an intriguing therapeutic avenue [45]. Similarly, the 1064 nm laser, which is effective for melasma treatment, can downregulate the PI3K/AKT/mTOR pathway, enhance melanocyte autophagy, and accelerate melanin metabolism [108]. Furthermore, understanding the role of exosomes in photoaging and their potential to restore skin physiological function by decreasing MMP expression and increasing collagen production could lead to novel exosome-based therapies for melasma [42].

### 6.5. Addressing Immune Dysregulation

The presence of subclinical inflammation suggests that immunomodulatory agents could play a role. While direct immune suppression might be too broad, understanding the specific inflammatory mediators and immune cell subsets involved could lead to targeted anti-inflammatory therapies. The role of mast cells, for instance, suggests that mast cell stabilizers or inhibitors of mast cell-derived mediators could be beneficial [61]. Indeed, oral ketotifen associated with famotidine can promote a slight improvement of facial melasma, providing evidence support [109].

### 6.6. Tranexamic Acid: A Multifaceted Agent

Tranexamic acid (TXA), a synthetic derivative of lysine, has emerged as an effective treatment for melasma in both females and males [110], acting through mechanisms that extend beyond simple melanogenesis inhibition [111]. TXA inhibits the plasminogen activator (PA) system, preventing the conversion of plasminogen to plasmin. UV exposure induces the keratinocyte-PA system, which in turn promotes melanogenesis. By inhibiting this system, TXA reduces melanin production. Moreover, TXA has been shown to prevent melanocyte multiplication and decrease tyrosinase activity, tyrosinase-related protein, and melanin content [111]. In an in vitro study, increased melanocore clusters are observed in TXA-treated keratinocytes, while the degradation of melanocores remains unchanged, indicating that TXA might affect the intracellular transport process of melanocores [112]. On the other hand, oral TXA also modulates systemic inflammation [113]. With high safety [114], its efficacy, whether oral [115], topical [116], or intradermal, highlights a therapeutic approach that targets a specific pathway (plasminogen/plasmin) that links UV exposure, inflammation, and melanogenesis, offering a comparative advantage by addressing a key upstream regulatory mechanism.

### 6.7. Holistic and Personalized Approaches

The complex pathogenesis of melasma necessitates a holistic and personalized approach to treatment. This involves not only targeting melanogenesis but also addressing dermal factors, inflammation, oxidative stress, and epidermal barrier dysfunction. Future research should focus on identifying specific melasma endotypes based on dominant pathogenic features (e.g., vascular-dominant, inflammatory-dominant, photoaging-dominant) to guide personalized therapeutic regimens [117]. The use of network pharmacology and molecular dynamics studies, as demonstrated by research on Cassipourea metabolites, can help identify potential multi-target agents that modulate genes and enzymes implicated in melasma pathogenesis, such as COX-2, TYR, ESR2, and ESR1, and pathways like prolactin and estrogen signaling [29]. This computational approach offers a powerful tool for discovering novel compounds with regulatory potential on multiple targets.

In conclusion, the shift in understanding melasma from a simple pigmentary disorder to a complex condition involving extensive epidermal–dermal crosstalk, chronic inflammation, and photoaging-related dysregulation provides a robust framework for developing more effective and durable treatments. By addressing the underlying microenvironmental abnormalities, novel therapeutic strategies can move beyond symptomatic relief to achieve more comprehensive and sustained improvement for patients suffering from this challenging disorder.

## 7. Conclusions

Melasma is a multifactorial disorder of the skin microenvironment characterized by epidermal–dermal crosstalk, immune dysregulation, vascular changes, and features of photoaging. This perspective highlights that melanocytes act within a broader pathological network rather than as isolated drivers of hyperpigmentation.

Future therapies should therefore extend beyond pigment suppression to target upstream processes such as fibroblast senescence, basement membrane disruption, angiogenesis, and chronic inflammation. By addressing these drivers in an integrated manner, more durable and patient-centered management of melasma can be achieved.

## Figures and Tables

**Table 1:** Genetic polymorphisms associated with melasma susceptibility.

Gene	Polymorphism/SNP	Population/Study	Association/Risk	Reference (Year)
*MC1R*	Val92Met (rs2228479)	Javanese women	Increased risk of melasma	Suryaningsih et al. (2019) [13]
*VDR*	TaqI (t allele/tt genotype)	Egyptian women	Associated with melasma	Seleit et al. (2017) [83]
*TYR*	rs1042602 (AA genotype)	African women (pilot)	Marked risk increase	Mpofana et al. (2025) [84]
*HERC2*	rs1129038	African women (pilot)	Population variation	Mpofana et al. (2025) [84]
*SLC24A5*	rs1426654	African women (pilot)	Population variation	Mpofana et al. (2025) [84]

**Table 2:** Main membrane proteins associated with intramelanosomal pH.

Results	Membrane Proteins	Function	Reference (Year)
Raise pH	SLC45A2	An H^+^-coupled glucose exporter in melanosomes	Liu et al. (2022) [98]
	OCA2	Outward transport of Cl^−^ from the melanosome lumen, which decreases the driving force for inward H^+^ transport by V-ATPase	Scales et al. (2021) [99]
Lower pH	V-ATPase	Pump the H^+^ into melanosomes in an ATP-dependent way	Collins et al. (2020) [100]
	TPC2	Generates membrane voltage (membrane potential) by the positive conductance, thus controlling the function of V-ATPase for H^+^ influx	Wiriyasermkul et al. (2020) [101]
	SLC24A5	Transport Ca2^+^ and K^+^ into the melanosome in exchange for Na^+^	Yousaf et al. (2021) [102]
	CLC7	As a 2Cl^−^/1H^+^ antiporter, pumping Cl^−^ into the lumen, which increases the V-ATPase driving force and acidifies the melanosome lumen	Koroma et al. (2021) [103]

## Data Availability

No new data were created or analyzed in this study. Data sharing is not applicable to this article.

## Abbreviations

The following abbreviations are used in this manuscript:

AC	adenylate cyclase
AMPK	AMP-activated protein kinase
bFGF	basic fibroblast growth factor
BM	basement membrane
cAMP	cyclic adenosine phosphate
Cer	ceramide
CGRP	calcitonin gene-related peptide
CLC7	chloride channel 7
COC	combined oral contraceptive
COX-2	cyclooxygenase-2
CREB	cAMP-response element binding protein
DDX5	DEAD-Box Helicase 5
ECM	extracellular matrix
EDN1	endothelin 1
EDNRB	endothelin receptor B
ERK	extracellular signal-regulated kinase
ESR	estrogen receptor
ET	endothelin
H2	histamine type 2
HEVL	high-energy visible light
HGF	hepatocyte growth factor
hIUD	hormonal intrauterine device
IGF	insulin-like growth factor
IL	interleukin
KGF	keratinocyte growth factor
LED	light-emitting diode
MAPK	mitogen-activated protein kinase
MC1R	melanocortin 1 receptor
MITF	microphthalm-associated transcription factor
MMP	matrix metalloproteinase
NEP	neural endopeptidase
NGF	nerve growth factor
NGFR	nerve growth factor receptor
OCA2	oculocutaneous albinism 2
OXT	oxytocin
PDL	pulsed-dye laser
PKA	protein kinase A
PKC	protein kinase C
POC	progestin-only contraceptive
ROS	reactive oxygen species
SASP	senescence-associated secretory phenotype
SCF	stem cell factor
SG	sebaceous gland
SLC	solute carrier
SNP	single-nucleotide polymorphism
TPC2	two-pore channel 2
TRPV	transient receptor potential vanilloid
TXA	tranexamic acid
TYR	tyrosinase
TYRP	tyrosinase-related protein
ULC	ultra-long chain
UV	ultraviolet
UVB	ultraviolet B
UVR	ultraviolet radiation
V-ATPase	vacuolar-type ATPase
VDR	vitamin D receptor
VEGF	vascular endothelial growth factor
VL	visible light
VLC	very long chain
αMSH	α-melanocyte stimulating hormone
